# Identification and characterization of human brown adipose tissue (BAT) content and metabolism in adults using [^18^F]-FDG PET/MR – a pilot study

**DOI:** 10.1186/2197-7364-1-S1-A68

**Published:** 2014-07-29

**Authors:** Helle H Johannesen, Johan Löfgren, Ida Donkin, Adam E Hansen, Annika Loft, Liselotte Højgaard, Andreas Kjær

**Affiliations:** Dept.of Clinical Physiology, Nuclear Medicine and PET, Rigshospitalet, University of Copenhagen, Kragujevac, Denmark; The Novo Nordisk Foundation Center for Basic Metabolic Research, Faculty of Health and Medical Sciences, University of Copenhagen, Kragujevac, Denmark

Brown adipose tissue (BAT) is present in human adults and when present visualised with [^18^F]-FDG PET. It contains large amounts of mitochondriae, which gives a higher water content compared to white adipose tissue and a high FDG avidity when activated [1].

Five adults underwent PET/MR. BAT was cold-activated using a water-perfused vest, with injection of 100Mbq FDG after 60 minutes [2]. Hereafter cervical regions were scanned on an Siemens Biograph mMR 3T. PET was performed as single bed 10 minutes acquisition.

MRI included DIXON sequence for attenuation correction and axial T2 weighted sequences with and without water suppression, the latter for calculation of water percentage (W%).

**Figure 1:**
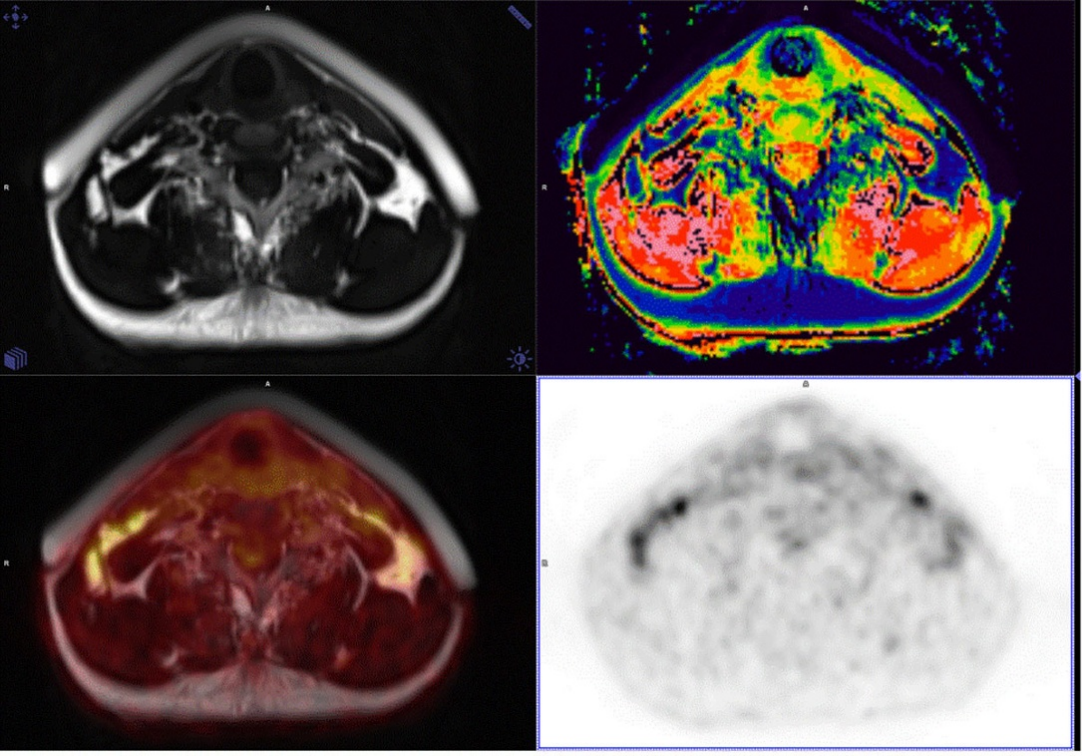
T2, water % , T2/PET and PET images.

ROIs were drawn on T2 images omitting vessels and muscle and propagated to PET images and to calculated water percentage maps (Figure 1). Subcutaneous fat regions were used as reference (SC).

**Figure 2:**
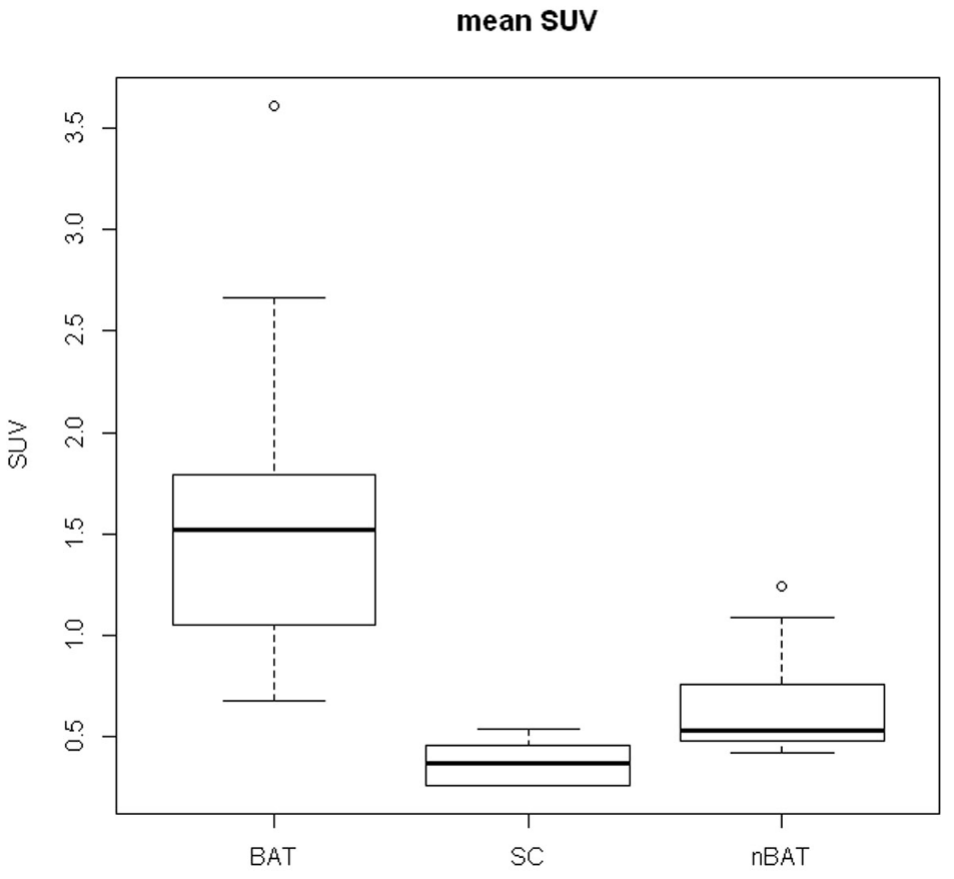
Box plot SUV value BAT, SC, nBAT

**Figure 3:**
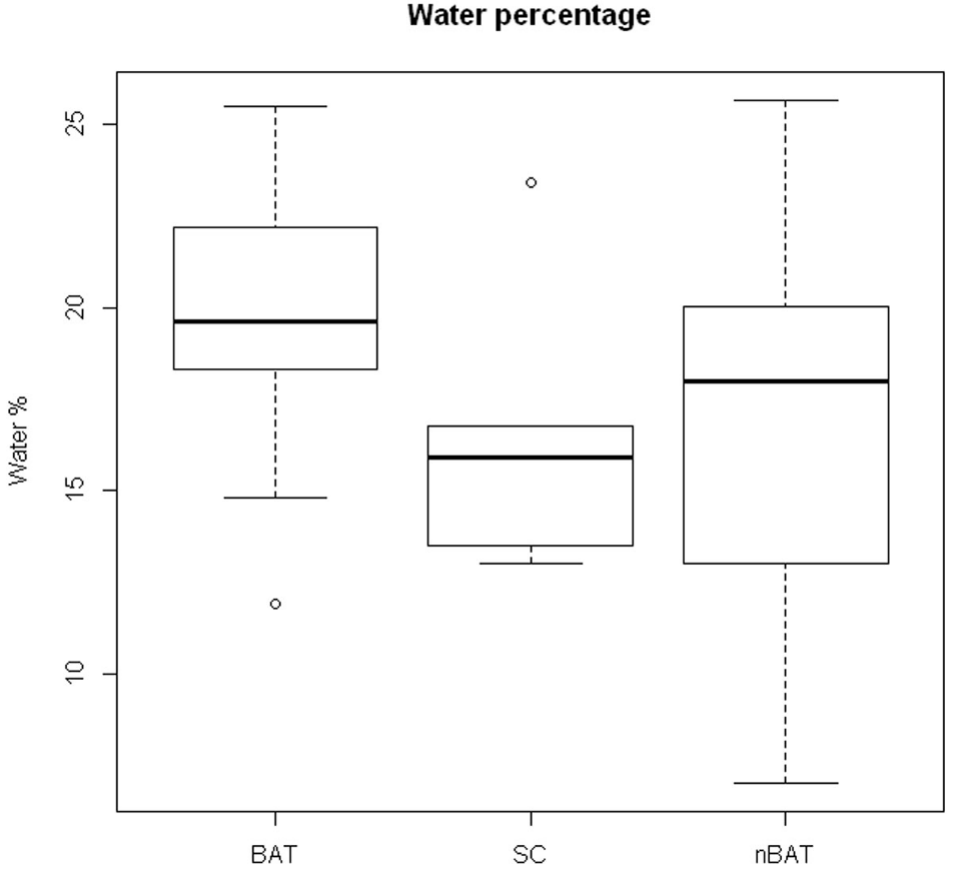
Box plot W% BAT, SC, nBAT

FDG-PET identified activated BAT in 3 of 5 subjects. On each scan eight ROIs were drawn, including one reference region (SC). SUVmean for BAT, nBAT and SC were 1.60 (SD 0.26), 0.38 ( 0.12) and 0.64 (0.12) respectively (Figure 2). Calculated water percentage MRI maps for these regions were 19.6% (SD2.9%), 16.5% (4.2%) and 16.8% (1.6%) respectively (Figure 3). No significant difference in W% was found between BAT and nBAT.

No significant correlation was found between water percentage in fat tissue with or without BAT, maybe because the two modalities measures different physiological properties. Accordingly, BAT can be present but not activated and therefore measurements with both modalities seems necessary for fully characterization of BAT.
